# Quantum simulation of Pauli channels and dynamical maps: Algorithm and implementation

**DOI:** 10.1371/journal.pone.0297210

**Published:** 2024-04-10

**Authors:** Tomás Basile, Carlos Pineda

**Affiliations:** 1 Facultad de Ciencias. Universidad Nacional Autónoma de México, Ciudad de México, Mexico; 2 Instituto de Física, Universidad Nacional Autónoma de México, Ciudad de México, México; Universita degli Studi Roma Tre, ITALY

## Abstract

Pauli channels are fundamental in the context of quantum computing as they model the simplest kind of noise in quantum devices. We propose a quantum algorithm for simulating Pauli channels and extend it to encompass Pauli dynamical maps (parametrized Pauli channels). A parametrized quantum circuit is employed to accommodate for dynamical maps. We also establish the mathematical conditions for an *N*-qubit transformation to be achievable using a parametrized circuit where only one single-qubit operation depends on the parameter. The implementation of the proposed circuit is demonstrated using IBM’s quantum computers for the case of one qubit, and the fidelity of this implementation is reported.

## 1 Introduction

Since their inception, quantum computers were proposed as powerful tools for the simulation of quantum systems [1]. Being open quantum systems of fundamental [2, 3] and practical [4] interest, there has been efforts towards the simulation of the evolution of open quantum systems [5–7] and specifically for quantum channels [8–11], which can be used to study and model decoherence. Such quantum algorithms can be represented using what is known as a quantum circuit [12], which we will study in section 3.

Such systems have been simulated because of their many applications, such as studying the emergence of multipartite entanglement [13, 14], studying dissipative processes [15] and modeling non-Markovian dynamics [16]. Among quantum systems, the simplest case is that of a qubit [12], and within them, the simplest class of channels that produce decoherence are Pauli channels [17–19]. Indeed, they serve as effective models for the noise affecting quantum devices [20].

In this article we propose a quantum algorithm for simulating Pauli channels and implement it on one of IBM’s quantum computers. The algorithm proposed is straightforward and can be used for any Pauli channel by only changing a couple of parameters in the operations it performs. To represent the algorithm, we use a quantum circuit, which is a common way of representing algorithms intended for quantum computers. [12]. Furthermore, we will also simulate Pauli dynamical maps, which are continuous parametrized curves of Pauli channels. These maps can be used to model a continuous change of a qubit instead of only discrete jumps. The generality of the algorithm proposed for Pauli channels will be very useful in this part, since then using for dynamical maps will come very naturally. Implementing these maps will lead us to study quantum algorithms with free parameters, something that is common in areas such as quantum machine learning [21]. This can be done using parametrized quantum circuits, which consist of quantum circuits in which one or more operations depend on a free parameter.

We start by providing the definition of quantum channels, the general framework used here, and multi-qubit Pauli channels in section 2. Our first objective is to present a general and straightforward quantum algorithm capable of simulating Pauli channels on quantum computers; we do this in section 3, where we also demonstrate its implementation using IBM’s quantum computers for several single and two-qubit Pauli channels. Expanding beyond Pauli channels, we introduce the concept of Pauli dynamical maps, defined as a continuous parametrization of multi-qubit Pauli channels. In fact, in order to implement them, we study parametrized quantum circuits in chapter 4. Furthermore, we contribute to the body of work related to parametrized quantum circuits by establishing a theorem, which sets the mathematical conditions for the transformations that can be done using a parametrized circuit with the restriction that only a controlled single-qubit rotation in the circuit may depend on the parameter. Finally, in section 5, we conclude analyzing the Pauli dynamical maps that fulfill the conditions of theorem 2.

## 2 Pauli channels and dynamical maps

In this section we introduce the concept of quantum channels, focusing on a specific type called Pauli channels. Furthermore, we define Pauli dynamical maps, which are curves of Pauli channels parametrized by a variable.

### 2.1 Quantum channels

In quantum mechanics, a closed system’s state is represented by a vector in a Hilbert space H. The state’s evolution is unitary and given by Schrodinger’s equation [22]. However, in real-world situations, quantum systems are usually open, which means that they interact with their environment [4]. For instance, the system’s state may become entangled with the environment, leading to a loss of information about the system’s state over time.

To describe open systems, instead of state vectors, we use matrices *ρ* that act on H. These matrices are called density matrices, and they include information about the system’s interaction with its environment. For a density matrix *ρ* to be physically valid, it must satisfy two conditions: tr(*ρ*) = 1 and it must be positive semi-definite, which is denoted as *ρ* ≥ 0 [12].

Knowing this, we can now define quantum channels. Quantum channels are operators E that can describe the evolution of open quantum systems, such that ρ→E(ρ). Quantum channels are the most general linear operations that a quantum system can undergo independently of its past [23, 24]. These channels are constructed based on three fundamental properties: linearity, trace preservation, and complete positivity.

Linearity ensures that a quantum channel E maps any ensemble of density matrices into the corresponding ensemble of their evolution. The trace preserving property is given by tr(E[ρ])=tr(ρ)=1 and guarantees that the quantum channel does not change the condition that tr(*ρ*) = 1. Finally, the channel should also preserve the condition *ρ* ≥ 0, and a map that does this is called a positive map. However, positivity of E is not enough, and we actually require the more restrictive condition of complete positivity. Complete positivity means that $E⊗I_{n}$ is positive for any positive integer *n* (where $I_{n}$ is the *n* × *n* identity matrix). This ensures that even if the main system is entangled with another system, applying E to the main system while doing nothing to the other one still results in a positive semidefinite state for the main system [12].

Given a quantum channel E, the condition of trace preservation is straightforward to verify but complete positivity is not as simple. To test complete positivity of a quantum channel, Jamiołkowski and Choi [25, 26] developed a simple algorithm that exploits the isomorphism between a channel E and the state D=(I⊗E)[|Ω〉〈Ω|], where $|\Omega \rangle =1/\text{dim}\left(H\right)\sum_{i}^{\text{dim}(H)}|i\rangle |i\rangle$ is a maximally entangled state between the original system and an ancilla. Remarkably, the map E is completely positive if and only if D (also known as the Choi or dynamical matrix of E) is positive semidefinite.

### 2.2 Pauli channels

We have discussed the main features of quantum channels and now we turn our attention to a specific type of channels for *N*-qubit systems called Pauli channels. First we will define these channels for single-qubit systems, whose most general density matrix can be written as [12]:

$$\begin{array}{c} \rho =\frac{1}{2}\sum_{\alpha =0}^{3}r_{\alpha }\sigma_{\alpha }, \end{array}$$ (1)

with $\sigma_{0}=I$, and *σ*_1,2,3_ the usual Pauli matrices. The condition tr(*ρ*) = 1 requires that *r*_0_ = 1 while *ρ* ≥ 0 implies that the remaining *r*_1,2,3_ form a vector $\vec{r}=\left(r_{1},r_{2},r_{3}\right)$ inside a unit sphere known as the Bloch sphere [27]. That is, every possible density matrix for a one-qubit system is uniquely associated with a point in a unit sphere.

Given a one-qubit system described by *ρ*, a Pauli channel is defined as an operation that with probability *k*_*γ*_ applies the Pauli matrix *σ*_*γ*_ to the system, for *γ* = 0, 1, 2, 3 [17]. Mathematically, the Pauli channel is written in the following way:

$$\begin{array}{c} E\left(\rho \right)=\sum_{\gamma =0}^{3}k_{\gamma }\sigma_{\gamma }\rho \sigma_{\gamma }, \end{array}$$ (2)

where the probabilities *k*_*γ*_ of applying *σ*_*γ*_ are non-negative real numbers such that ∑_*γ*_ *k*_*γ*_ = 1 (these conditions also ensure that the channel is trace preserving and completely positive).

Pauli channels are some of the most fundamental noise models in quantum information science [28]. Some notable examples of Pauli channels are the following:

- **Bit Flip Channel:** This is a channel that with probability 1 − *p* leaves the qubit as it is and with probability *p* applies the *σ*_1_ matrix (which flips the basis states |0〉 and |1〉 of the qubit), and so it is given by:
  $$\begin{array}{c} E\left(\rho \right)=\left(1-p\right)\rho +p\sigma_{1}\rho \sigma_{1}. \end{array}$$
  Analogous channels exist using *σ*_3_ (called the bit flip channel, which has a probability *p* of adding a relative phase *π* to the state) or using *σ*_2_ (called the phase flip channel, which has a probability *p* of flipping the base states and also add a relative phase *π*).
- **Depolarizing channel:** This channel has a probability 1 − *p* of doing nothing to the qubit and a probability *p* of converting it into the maximally mixed state $\frac{1}{2}I$ and it can be written as:
  $$\begin{array}{c} E\left(\rho \right)=\left(1-p\right)\rho +p\frac{1}{2}I=(1-\frac{3p}{4})\sigma +\frac{p}{4}\sigma_{1}\rho \sigma_{1}+\frac{p}{4}\sigma_{2}\rho \sigma_{2}+\frac{p}{4}\sigma_{3}\rho \sigma_{3}. \end{array}$$ (3)

We can also see how an arbitrary Pauli channel acts on an arbitrary density matrix. To do it, we substitute Eq (1) in Eq (2):

$$\begin{array}{c} E\left(\rho \right)=\frac{1}{2}\sum_{\gamma ,\alpha =0}^{3}k_{\gamma }r_{\alpha }\sigma_{\gamma }\sigma_{\alpha }\sigma_{\gamma }. \end{array}$$ (4)

This can be simplified noting that

$$\begin{array}{c} \sigma_{\gamma }\sigma_{\alpha }\sigma_{\gamma }=A_{\alpha ,\gamma }\sigma_{\alpha },\;\;\;\;\;\text{with}\;A=(\begin{array}{cccc} 1 & 1 & 1 & 1\\ 1 & 1 & -1 & -1\\ 1 & -1 & 1 & -1\\ 1 & -1 & -1 & 1 \end{array}), \end{array}$$ (5)

which was obtained following the commutation relation of Pauli matrices [*σ*_*α*_, *σ*_*β*_] = 2*iϵ*_*αβγ*_*σ*_*γ*_ and *ϵ*_*αβγ*_ is the Levi-Civita tensor. Then, Eq (4) takes the form

$$\begin{array}{c} E\left(\rho \right)=\frac{1}{2}\sum_{\alpha }(\sum_{\gamma }A_{\alpha ,\gamma }k_{\gamma })r_{\alpha }\sigma_{\alpha }. \end{array}$$ (6)

Eq (6) once again has the form of Eq (1) but with components (∑_*γ*_ *A*_*α*,*γ*_*k*_*γ*_)*r*_*α*_. This gives us another way of understanding Pauli channels as operations that take each component *r*_*α*_ of the density matrix and multiplies them by ∑_*γ*_ *A*_*α*,*γ*_*k*_*γ*_, that is:

$$\begin{array}{c} r_{\alpha }\overset{\text{Pauli}}{\underset{\text{Channel}}{\to }}\tau_{\alpha }r_{\alpha },\;\;\tau_{\alpha }≔\sum_{\gamma }A_{\alpha ,\gamma }k_{\gamma }. \end{array}$$ (7)

Notice that *τ*_0_ = 1, which is a consequence of ∑_*γ*_ *k*_*γ*_ = 1 and ensures that after the channel, the resulting density matrix still has trace one. Furthermore, reverting the definition of *τ*_*α*_ by using that $A^{-1}=\frac{1}{4}A$, we get that $k_{\gamma }=\frac{1}{4}\sum_{\alpha }A_{\alpha ,\gamma }\tau_{\alpha }$. Then, using that *k*_*γ*_ ≥ 0 we get the following conditions on the multipliers *τ*_*α*_:

$$\begin{array}{c} 1+\tau_{i}-\tau_{j}-\tau_{k}\geq 0,\;\;\;\text{for}\;i,j,k\;\text{different}\;\text{numbers}\;\text{in}\;\left\{1,2,3\right\}, \end{array}$$ (8)

$$\begin{array}{c} 1+\tau_{1}+\tau_{2}+\tau_{3}\geq 0. \end{array}$$ (9)

These conditions imply that (*τ*_1_, *τ*_2_, *τ*_3_) has to be inside a tetrahedron with vertices (1, 1, 1), (1, −1, −1), (−1, 1, −1) and (−1, −1, 1). Therefore, the *τ*_1,2,3_ are numbers between −1 and 1, which means that the components *r*_*α*_ of the density matrix are always attenuated and possibly sign flipped.

Having defined the one qubit case, we can now generalize to *N* qubits. In order to do it, we need to introduce the so-called *Pauli strings*, defined as

$$\begin{array}{c} \sigma_{\vec{\alpha }}=\sigma_{\alpha_{1}}⊗\sigma_{\alpha_{2}}⊗\cdots ⊗\sigma_{\alpha_{N}}, \end{array}$$ (10)

where $\vec{\alpha }$ denotes a multi-index (*α*_1_, ⋯, *α*_*N*_) and *α*_*i*_ ∈ {0, 1, 2, 3}. These operators form an orthogonal basis in the space of operators acting on *N* qubits. Similarly to the single-qubit case, the density matrix *ρ* of a system of *N* qubits can be written using Pauli strings as:

$$\begin{array}{c} \rho =\frac{1}{2^{N}}\sum_{\vec{\alpha }}r_{\vec{\alpha }}\sigma_{\vec{\alpha }}. \end{array}$$ (11)

Then, just as before, we define a Pauli channel as a transformation that applies the operator $\sigma_{\vec{\gamma }}$ to *ρ* with probability $k_{\vec{\gamma }}$ and is therefore described mathematically by:

$$\begin{array}{c} E\left(\rho \right)=\sum_{\vec{\gamma }}k_{\vec{\gamma }}\sigma_{\vec{\gamma }}\rho \sigma_{\vec{\gamma }}, \end{array}$$ (12)

where just as before, $k_{\vec{\gamma }}$ are non-negative real numbers such that $\sum_{\vec{\gamma }}k_{\vec{\gamma }}=1$.

As in the one qubit case, Pauli channels for *N* qubits attenuate the components $r_{\vec{\alpha }}$ of the density matrix. This can be seen by substituting Eq (11) in Eq (12) and using the property of Eq (5):

$$\begin{array}{cc} E(\rho ) & =\frac{1}{2^{N}}\sum_{\vec{\gamma },\vec{\alpha }}k_{\vec{\gamma }}r_{\vec{\alpha }}\sigma_{\vec{\gamma }}\sigma_{\vec{\alpha }}\sigma_{\vec{\gamma }}\\ & =\frac{1}{2^{N}}\sum_{\vec{\alpha }}(\sum_{\vec{\gamma }}{\left(A^{{⊗}^{N}}\right)}_{\vec{\alpha },\vec{\gamma }}\;k_{\vec{\gamma }})\;r_{\vec{\alpha }}\sigma_{\vec{\alpha }}, \end{array}$$

which means that applying the Pauli channel multiplies the components $r_{\vec{\alpha }}$ by $\tau_{\vec{\alpha }}≔\sum_{\vec{\gamma }}{\left(A^{{⊗}^{N}}\right)}_{\vec{\alpha },\vec{\gamma }}k_{\vec{\gamma }}$.

### 2.3 Pauli dynamical maps

As seen in the last section, Pauli channels and in general quantum channels are discrete maps that transform a density matrix *ρ* into E(ρ). However, we could also define a continuous set of channels *ε*_*p*_ with *p* a real parameter.

For the special case of Pauli channels, we define a Pauli dynamical map as a continuous parametrized curve drawn inside the set of Pauli channels and starting at the identity channel. Therefore, a Pauli dynamical map can be written as

$$\begin{array}{c} E_{p}\left(\rho \right)=\sum_{\vec{\gamma }}k_{\vec{\gamma }}\left(p\right)\sigma_{\vec{\gamma }}\rho \sigma_{\vec{\gamma }}, \end{array}$$ (13)

where *p* is a parameter in an interval [*a*, *b*] and $E_{p}$ is a Pauli channel for every *p*, with $E_{a}$ being the identity channel.

## 3 Circuit for a Pauli channel

In this section we propose a quantum circuit that simulates *N*-qubit Pauli channels. A quantum circuit is a model for representing computation on a quantum system. This computation may include preparing the initial state of the system, applying unitary operations and measuring the whole system or parts of it [22]. Our systems always consists of some number of qubits, that can be divided into the ones to which we will apply the quantum channel, or main qubits and the ones that help us complete said operation, or ancilla qubits. The circuit implements a unitary operator *U* on the system consisting of all the qubits and then the effect on the main qubits is measured. Moreover, we implement the circuit for *N* = 1 on a quantum computer and analyze the results using the diamond norm. We find that close to the depolarizing channel, the general circuit simulator can implement such channels with the highest fidelity.

### 3.1 Description of the circuit for a Pauli channel

**Fig 1:**
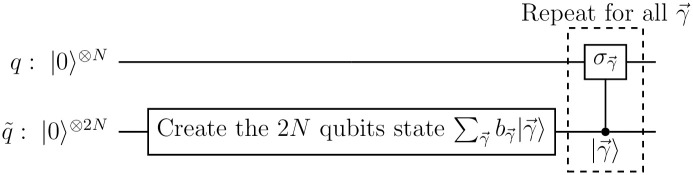
Circuit for an *N*-qubit Pauli channel. The circuit creates the state $\sum_{\vec{\gamma }}b_{\vec{\gamma }}|\vec{\gamma }\rangle$ on the 2*N* ancilla qubits denoted by $\tilde{q}$.

To design the circuit that implements Eq (12), we construct a state that includes the probabilities *k*_*γ*_ on the ancilla qubits and subsequently apply controlled Pauli operations on the main qubits. The circuit that does this is presented in Fig 1.

The first part of the circuit involves the creation of the state

$$\begin{array}{c} \text{Ancilla}\;\text{state}\;=\sum_{\vec{\gamma }}b_{\vec{\gamma }}\left|\vec{\gamma }\right\rangle \end{array}$$ (14)

on the ancilla qubits, where $b_{\vec{\gamma }}$ are numbers such that ${|b_{\vec{\gamma }}|}^{2}=k_{\vec{\gamma }}$ and the 2*N*-qubit state $|\vec{\gamma }\rangle$ is defined as |*γ*_1_〉⋯|*γ*_*N*_〉. When measured in the computational basis, the state given in Eq (15) collapses to $|\vec{\gamma }\rangle$ with a probability ${|b_{\vec{\gamma }}|}^{2}=k_{\vec{\gamma }}$. The circuit in Fig 1 uses this fact to apply $\sigma_{\vec{\gamma }}$ on the main qubits with a probability $k_{\vec{\gamma }}$ by using controlled operations conditioned on the state of the system being $|\vec{\gamma }\rangle$, just as the Pauli channel is supposed to do.

### 3.2 Simulation for one-qubit Pauli channels

**Fig 2:**
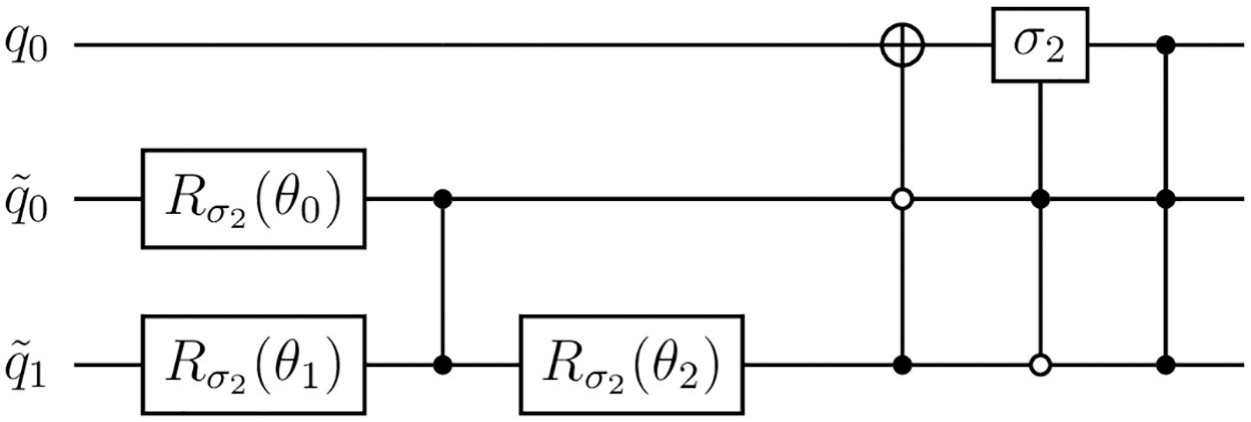
One-qubit Pauli channel circuit. Circuit for a one-qubit Pauli channel, which is a particular case of Fig 1. Here we have two ancilla qubits and we use three rotations of angles given by Eq (15) to create the Ancilla State of of Eq (14) for the two ancilla qubits.

For the particular case of a Pauli channel on one qubit, the circuit that simulates it can be constructed as in Fig 2, which is a special case of Fig 1 but with all details explicitly shown. In said figure, the ancilla state of Eq (14) can be taken to be $\sqrt{k_{0}}|00\rangle +\sqrt{k_{1}}|01\rangle +\sqrt{k_{2}}|10\rangle +\sqrt{k_{3}}|11\rangle$ and it is created on the ancilla qubits with the help of three rotations of angles defined by the following equations:

$$\begin{array}{cc} \text{cos}\;(\frac{\theta_{0}}{2}) & =\sqrt{k_{0}+k_{1}},\\ \text{tan}\;(\frac{\theta_{1}+\theta_{2}}{2}) & =\sqrt{k_{1}/k_{0}},\\ \text{tan}\;(\frac{\theta_{2}-\theta_{1}}{2}) & =\sqrt{k_{3}/k_{2}}. \end{array}$$ (15)

**Fig 3:**
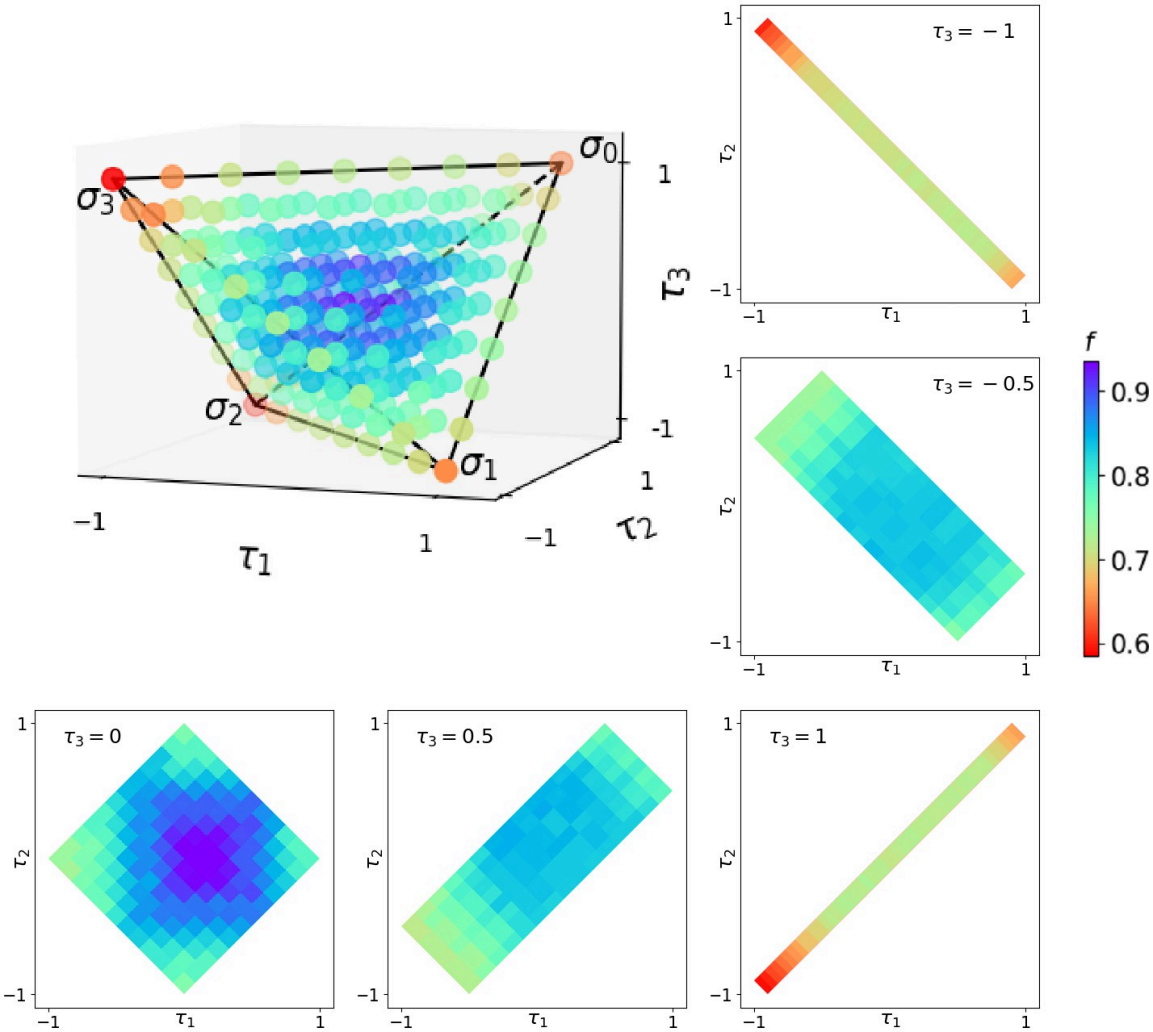
Results of the diamond fidelities as defined in Eq (18) for a sample of Pauli channels in the tetrahedron. Notice that channels close to the center of the tetrahedron have high fidelities, while those close to the borders do not. Moreover, we show the results for cuts of the tetrahedron at different values of *τ*_3_.

We took a sample of one-qubit Pauli channels and evaluated their implementation on IBM’s ibmq-lima quantum computer [29], as shown in Fig 3. For each of the channels sampled, we used quantum process tomography [29, 30] to obtain the operator *ξ*_*I*_ corresponding to the implementation of the circuit in the quantum computer. Then, we compared *ξ*_*I*_ with the theoretical operator *ξ*_*T*_ of the Pauli channel we wanted to implement. To see how close the operators *ξ*_*I*_ and *ξ*_*T*_ are, we shall use the diamond distance [31], which is defined by

$$\begin{array}{c} \left|\right|\xi_{I}-\xi_{T}{\left|\right|}_{◇}=\underset{\rho }{\text{max}}\;\left|\right|\left(\xi_{I}⊗I\right)\rho -\left(\xi_{T}⊗I\right)\rho {\left|\right|}_{1}, \end{array}$$ (16)

with *I* the identity map, || ⋅ ||_1_ the trace norm and the maximization done over all density matrices *ρ*. The calculation of this norm is done using the semi-definite program from reference [32]. When the two channels are the same, the diamond distance has a value of 0, while in the case that the channels are completely distinguishable, the distance reaches its maximum value of 2 [33]. For the analysis done in Fig 3, we define a sort of “diamond fidelity” as:

$$\begin{array}{c} f=1-\frac{1}{2}\left|\right|\varepsilon_{I}-\varepsilon_{T}{\left|\right|}_{◇}, \end{array}$$ (17)

which ranges from 0, when the channels have a maximum distance, to 1, when they are exactly equal.

Finally, using the representation of Pauli channels in a tetrahedron as in Eq (8), we show in Fig 3 the diamond fidelity defined by Eq (17) for the channels analyzed. We can see that channels close to the completely depolarizing channel (that is close to the center of the tetrahedron) have a high *f*, while those close to unitary channels have much lower *f*. This is reasonable because quantum computers are prone to errors that depolarize qubits, which isn’t very problematic when trying to simulate depolarization but it is when simulating unitary processes. Moreover, as can be seen in Fig 3, the algorithm of Fig 2 is not optimal for channels in the edges of the tetrahedron, which corresponds to unitary channels. These channels could be accomplished more efficiently by simply applying the corresponding unitary operator directly. In this case we would be trading generality for fidelity. The main advantage of the algorithm is that we can simulate with a unitary circuit any Pauli channel, only varying a couple of angles in the circuit. In fact, as we will see, sometimes only changing one angle in the circuit will be enough to simulate many non-unitary dynamical maps of physical interest.

### 3.3 Simulation of a two-qubit Pauli channel

**Fig 4:**
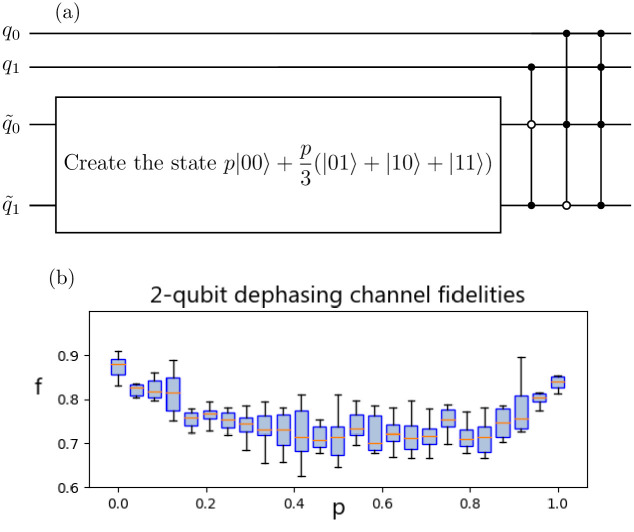
Circuit for 2-qubit dephasing channel. Circuit implementation for the 2-qubit dephasing channel of Eq (17) and fidelity results of its implementation using ibmq-lima. The circuit is shown in figure a), where the state on the two ancilla qubits can be constructed just as it was done for Fig 2. In figure b) we show the results of fidelities after implementing this circuit on ibmq-lima for 25 values of *p* between 0 and 1, each of which is simulated 10 times.

For the general case of a two-qubit Pauli channel, the circuit of Fig 1 requires two main qubits and four ancilla qubits. However, the quantum computer which we are using has only 5 qubits. We could still simulate a two qubit Pauli channel that applies at most 8 Pauli operations, instead of 16 which would be the most general case, using the three ancilla qubits. For example, consider the following two-qubit dephasing channel:

$$\begin{array}{c} \varepsilon \left(\rho \right)=\left(1-p\right)\rho +\frac{p}{3}\left(I⊗\sigma_{z}\right)\rho \left(I⊗\sigma_{z}\right)+\frac{p}{3}\left(\sigma_{z}⊗I\right)\rho \left(\sigma_{z}⊗I\right)+\frac{p}{3}\left(\sigma_{z}⊗\sigma_{z}\right)\rho \left(\sigma_{z}⊗\sigma_{z}\right), \end{array}$$ (18)

where *p* ∈ [0, 1] is a parameter that defines the channel. Since this channel has four Pauli operations, only 2 ancilla qubits will be necessary, and thus it can be implemented using the circuit of Fig 4b.

Just as for the one-qubit Pauli channels, this channel was implemented on IBM’s ibmq-lima quantum computer. We obtained the Choi matrix by doing quantum process tomography on the main qubits. Then, we calculated the diamond fidelity for different values of *p* and obtained the results shown in Fig 4a. As can be seen in the figure, the fidelity doesn’t vary much as we change the value of *p*. The average fidelity over all values of *p* is 0.758 and it is biggest when *p* = 0, corresponding to the identity channel, where it reaches a fidelity of 0.874.

## 4 One parameter circuits

Finally, we consider the simulation of Pauli dynamical maps. This is pretty much already solved, since just as Pauli channels, Pauli dynamical maps can be implemented using the circuit of Fig 1. However, there is one difference: the state to be created on the ancilla qubits now depends on a parameter *p*, and it is represented by the expression:

$$\begin{array}{c} \sum_{\vec{\gamma }}b_{\vec{\gamma }}\left(p\right)\left|\vec{\gamma }\right\rangle . \end{array}$$ (19)

Thus, we temporarily shift our focus from Pauli channels and dynamical maps to the general problem of creating a circuit to generate a curve of states like the one described in Eq (19).

In general, producing this curve of states for *N* qubits will require many rotations parametrized by *p*, such as the three rotations used for the ancilla qubits in Fig 2. However, it would be preferable to achieve the same effect using only one parametrized rotation. This would allow us to interpret said rotation as a knob that smoothly traverses the curve of states. Consequently, we are faced with the question of which curves of states, such as the one described in Eq (19), can be produced using just a single parametrized rotation. To clarify this, we provide the following definition for a circuit with one parametrized rotation.

**Definition 1** ***1-Parameter Rotation Circuit:*** *A 1-Parameter Rotation (1PR) circuit is a parametrized quantum circuit that includes only one gate dependent on a parameter p. Moreover, the parametrized gate is a one-qubit rotation about any axis, whether controlled or not*.

Therefore, a 1PR circuit implements a unitary operator *U*(*p*) on some number *N* of qubits, such that *U* depends on the parameter *p* only locally on one specific qubit.

**Fig 5:**
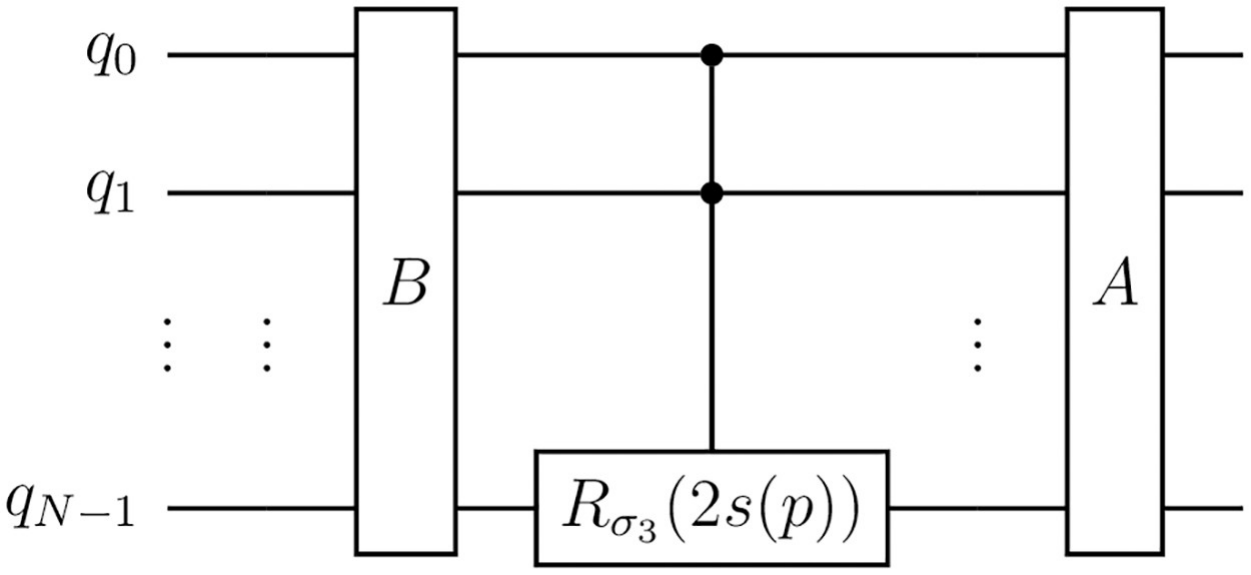
General form of a 1PR circuit. Any 1PR circuit can be transformed into this form, where the rotation on the last qubit can be controlled or not by any of the other qubits. *A* and *B* are *N*-qubit gates that do not depend on the parameter *p* and *s* = *s*(*p*) is a function of the parameter.

Based on this definition, we aim to determine which curves of states can be generated using 1PR circuits. To accomplish this, we begin by proving that all 1PR circuits have the form depicted in Fig 5, where the parametrized rotation is around *σ*_3_ and is applied to the last qubit.

**Theorem 1** *An N* − *qubit 1PR circuit can always be transformed into the form shown in* Fig 5.

**Proof:** First, we observe that according to the definition, a 1PR circuit always consists of an operation *B* followed by the parametrized rotation on a specific qubit and then another operation *A*, where *A* and *B* are not parametrized.

Next, we note that it is not necessary to consider rotations about an arbitrary axis, as a rotation about any axis $\hat{n}$ parameterized by *p* can be transformed into a rotation about *σ*_3_ without introducing gates that depend on *p*. To see this, consider the rotation $R_{\hat{n}}\left(2s\right)$, where 2*s* is a function of *p* (the factor of 2 is for convenience later on) and $\hat{n}=\left(n_{1},n_{2},n_{3}\right)$ represents the rotation axis. We can express $\hat{n}$ as (sin *θ* cos *ϕ*, sin *θ* sin *ϕ*, cos *θ*), where *θ* and *ϕ* are fixed angles dependent on $\hat{n}$. The rotation can then be rewritten as follows:

$$\begin{array}{c} R_{\hat{n}}\left(2s\right)=R_{\sigma_{3}}\left(\phi \right)R_{\sigma_{2}}\left(\theta \right)R_{\sigma_{3}}\left(2s\right)R_{\sigma_{2}}\left(-\theta \right)R_{\sigma_{3}}\left(-\phi \right). \end{array}$$ (20)

Since the angles *θ* and *ϕ* do not depend on the parameter *p*, any 1PR circuit can be transformed into a circuit where the parametrized rotation is around *σ*_3_ instead of an arbitrary axis. Moreover, without loss of generality, we can choose the last qubit as the target qubit for the rotation, since if it is not, we could use swap gates to move the rotation to the first qubit without adding gates that depend on *p*.

Therefore, a 1PR circuit can be transformed such that the rotation is around *σ*_3_ and is applied to the last qubit (possibly controlled by other qubits), resulting in the form depicted in Fig 5.

With the aid of this theorem, we can now determine the curves of states of *N* qubits that can be generated using a 1PR circuit. This result is stated in the following theorem.

**Theorem 2** *Consider a 1PR circuit of N qubits parametrized by p and denote by U*(*p*) *the operator it implements on this system. Then, for every j* ∈ {0, 1, ⋯, 2^*N*^ − 1}, *we have that*:

$$\begin{array}{c} U\left(p\right)|j\rangle =e^{is(p)}|a^{j}\rangle +e^{-is(p)}|b^{j}\rangle +|c^{j}\rangle \end{array}$$ (21)

*with s*(*p*) *some function of p*, |*a*^*j*^〉, |*b*^*j*^〉, |*c*^*j*^〉 *orthogonal states and* 〈*a*^*j*^|*a*^*j*^〉 + 〈*b*^*j*^|*b*^*j*^〉 + 〈*c*^*j*^|*c*^*j*^〉 = 1. *Here* |*j*〉 *represents an element of the N-qubit computational basis*.

**Proof:** As before, from the definition of a 1PR circuit, we know that *U*(*p*) can be decomposed as *ARB*, where *A*, *B* are unitary operators acting on the *N*-qubit system and *R* is a rotation applied to one qubit. From theorem 1, we conclude that *R* can be taken in general as a *σ*_3_ rotation of angle 2*s*(*p*) applied to the last qubit and controlled by some of the other ones. To prove this theorem, we will apply *U*(*p*) to |*j*〉 by first applying *B*, then $R_{\sigma_{3}}\left(2s\left(p\right)\right)$ and finally *A*, with the goal of getting to Eq (21).

First, applying *B* to |*j*〉 results in $B|j\rangle =B_{0,j}|0\rangle +B_{1,j}|1\rangle +\cdots +B_{2^{n}-1,j}|2^{n}-1\rangle$, with *B*_*i*,*j*_ the entries of matrix *B*. This can be rewritten by separating the last qubit from the other *N* − 1:

$$\begin{array}{c} B|j\rangle =\sum_{k=0}^{2^{N-1}-1}(B_{2k,j}\left|k\rangle |0\right\rangle +B_{2k+1,j}\left|k\rangle |1\right\rangle ). \end{array}$$ (22)

After the operator *B*, the circuit applies the controlled rotation $R_{\sigma_{3}}\left(2s\left(p\right)\right)$. To simplify the analysis, we separate the states of the first *N* − 1 qubits into those that fulfill the control conditions of the rotation (which we denote as the set C) and those that do not, and write it as

$$\begin{array}{c} B|j\rangle =\sum_{k\in C}(B_{2k,j}\left|k\rangle |0\right\rangle +B_{2k+1,j}\left|k\rangle |1\right\rangle )+\sum_{k\notin C}(B_{2k,j}\left|k\rangle |0\right\rangle +B_{2k+1,j}\left|k\rangle |1\right\rangle ). \end{array}$$ (23)

Then, the rotation *R* will only affect the states on the first sum (since they fulfill the control conditions) and not the others. Therefore, using that a $R_{\sigma_{3}}\left(2s\left(p\right)\right)$ rotation acts by adding a phase *e*^−*is*(*p*)^ to |0〉 and a phase *e*^*is*(*p*)^ to |1〉, we have that,

$$\begin{array}{cc} R_{\sigma_{3}}\left(2s\left(p\right)\right)B\left|j\right\rangle & =e^{-is(p)}\sum_{k\in C}B_{2k,j}\left|k\rangle |0\right\rangle +e^{is(p)}\sum_{k\in C}B_{2k+1,j}\left|k\rangle |1\right\rangle +\sum_{k\notin C}(B_{2k,j}\left|k\rangle |0\right\rangle +B_{2k+1,j}\left|k\rangle |1\right\rangle )\\ & =e^{-is(p)}|{\tilde{b}}^{j}\rangle +e^{is(p)}|{\tilde{a}}^{j}\rangle +|{\tilde{c}}^{j}\rangle , \end{array}$$ (24)

where we defined

$$\begin{array}{cc} |{\tilde{a}}^{j}\rangle & =\sum_{k\in C}B_{2k,j}|k\rangle |1\rangle ,\\ |{\tilde{b}}^{j}\rangle & =\sum_{k\in C}B_{2k+1,j}|k\rangle |0\rangle ,\\ |{\tilde{c}}^{j}\rangle & =\sum_{k\notin C}(B_{2k,j}\left|k\rangle |0\right\rangle +B_{2k+1,j}\left|k\rangle |1\right\rangle ). \end{array}$$

These states are clearly orthogonal because they are each linear combinations of different orthogonal states of the computational basis. Moreover, they satisfy 〈*a*^*j*^|*a*^*j*^〉 + 〈*b*^*j*^|*b*^*j*^〉 + 〈*c*^*j*^|*c*^*j*^〉 = 1 because this quantity is the squared norm of the *j*th column of *B*, which is unitary.

Finally, after having applied the rotation, the circuit applies gate *A*, so that the result is given by:

$$\begin{array}{cc} U|j\rangle & =AR_{\sigma_{3}}\left(2s\left(p\right)\right)B\left|j\right\rangle \\ & =e^{-is(p)}A|{\tilde{a}}^{j}\rangle +e^{is(p)}A|{\tilde{b}}^{j}\rangle +A|{\tilde{c}}^{j}\rangle \\ & =e^{-is(p)}|a^{j}\rangle +e^{is(p)}|b^{j}\rangle +|c^{j}\rangle , \end{array}$$ (25)

where $|a^{j}\rangle =A|{\tilde{a}}^{j}\rangle ,|b^{j}\rangle =A|{\tilde{b}}^{j}\rangle ,|c^{j}\rangle =A|{\tilde{c}}^{j}\rangle$ are still orthogonal states that satisfy 〈*a*|*a*〉 + 〈*b*|*b*〉 + 〈*c*|*c*〉 = 1 because *A* is unitary.

This theorem implies that when starting from the state |0〉 or any other initial state, the only possible curves of states that can be created using a 1PR circuit are of the following form:

$$\begin{array}{c} \left|\eta \left(p\right)\right\rangle =\left|c\right\rangle +e^{is(p)}\left|a\right\rangle +e^{-is(p)}\left|b\right\rangle , \end{array}$$ (26)

with conditions defined by the equations:

$$\begin{array}{c} \langle a|a\rangle +\langle b|b\rangle +\langle c|c\rangle =1,\;\langle a|b\rangle =\langle a|c\rangle =\langle b|c\rangle =0. \end{array}$$ (27)

Moreover, it is possible to construct a 1PR circuit to generate any given curve of states described by Eq (26). One approach to achieve this is by utilizing the circuit depicted in Fig 5, with the parametrized rotation *R* applied to the last qubit controlled by all the other qubits. There is some freedom when choosing the operators *A* and *B*, we only need to make sure that:

$$\begin{array}{c} B|0\rangle =\sqrt{\left\langle a|a\right\rangle }|2^{N}-1\rangle +\sqrt{\left\langle b|b\right\rangle }|2^{N}-2\rangle +\sqrt{\left\langle c|c\right\rangle }\left|2^{N}-3\right\rangle ,\\ A|2^{N}-1\rangle =\frac{1}{\sqrt{\left\langle a|a\right\rangle }}\left|a\rangle ,\;A\right|2^{N}-2\rangle =\frac{1}{\sqrt{\left\langle b|b\right\rangle }}\left|b\rangle ,\;A\right|2^{N}-3\rangle =\frac{1}{\sqrt{\left\langle c|c\right\rangle }}\left|c\right\rangle . \end{array}$$ (28)

The remaining part of the operators *A* and *B* can be chosen in any arbitrary manner as long as they are unitary. Examples of these matrices are shown for particular dynamical maps in the next section.

To see that $AR_{\sigma_{3}}\left(s\left(p\right)\right)B|0\rangle$ in fact creates the curve of states described in Eq (26), we can rewrite the expression of *B*|0〉 by separating the first *N* − 1 qubits from the last one:

$$B|0\rangle =\sqrt{\left\langle a|a\right\rangle }|2^{N-1}-1\rangle |1\rangle +\sqrt{\left\langle b|b\right\rangle }|2^{N-1}-1\rangle |0\rangle +\sqrt{\left\langle c|c\right\rangle }|2^{N-1}-2\rangle |1\rangle .$$

Since the parametrized rotation $R_{\sigma_{3}}\left(2s\left(p\right)\right)$ is controlled by all the first *N* − 1 qubits, it only applies to the first two terms of *B*|0〉. As a result, we obtain:

$$R_{\sigma_{3}}\left(2s\left(p\right)\right)B|0\rangle =\sqrt{\left\langle a|a\right\rangle }e^{is(p)}|2^{N-1}-1\rangle |1\rangle +\sqrt{\left\langle b|b\right\rangle }e^{-is(p)}|2^{N-1}-1\rangle |0\rangle +\sqrt{\left\langle c|c\right\rangle }|2^{N-1}-2\rangle |1\rangle .$$

Finally, applying the operator *A* to this state yields:

$$\begin{array}{c} AR_{\sigma_{3}}\left(2s\left(p\right)\right)B=\left|c\right\rangle +e^{is(p)}\left|a\right\rangle +e^{-is(p)}\left|b\right\rangle . \end{array}$$

With this, we prove that any curve of states as that in Eq (26) can be constructed by the circuit in Fig 5 with the parametrized rotation controlled by all the other qubits by correctly choosing matrices *A* and *B*.

## 5 1PR circuit for a Pauli map

We can now use the previous results to conclude directly which Pauli dynamical maps can be implemented with a 1PR circuit. For this, the curve of states of Eq (19) has to be constructed with only one parametrized rotation, so it has to satisfy the conditions of theorem 2. Therefore, this implies that the map

$$\begin{array}{c} \varepsilon_{p}\left(\rho \right)=\sum_{\vec{\gamma }}k_{\vec{\gamma }}\left(p\right)\sigma_{\vec{\gamma }}\rho \sigma_{\vec{\gamma }}, \end{array}$$ (29)

can be implemented if there are numbers $\beta_{\vec{\gamma }}\left(p\right)$ such that $|\beta_{\vec{\gamma }}{\left(p\right)|}^{2}=k_{\vec{\gamma }}\left(p\right)$ and

$$\begin{array}{c} \sum_{\vec{\gamma }}\beta_{\vec{\gamma }}\left(p\right)|\vec{\gamma }\rangle =|c\rangle +e^{is(p)}\left|a\right\rangle +e^{-is(p)}\left|b\right\rangle , \end{array}$$ (30)

where |*a*〉, |*b*〉, |*c*〉 fulfill the conditions of Eq (27).

**Fig 6:**
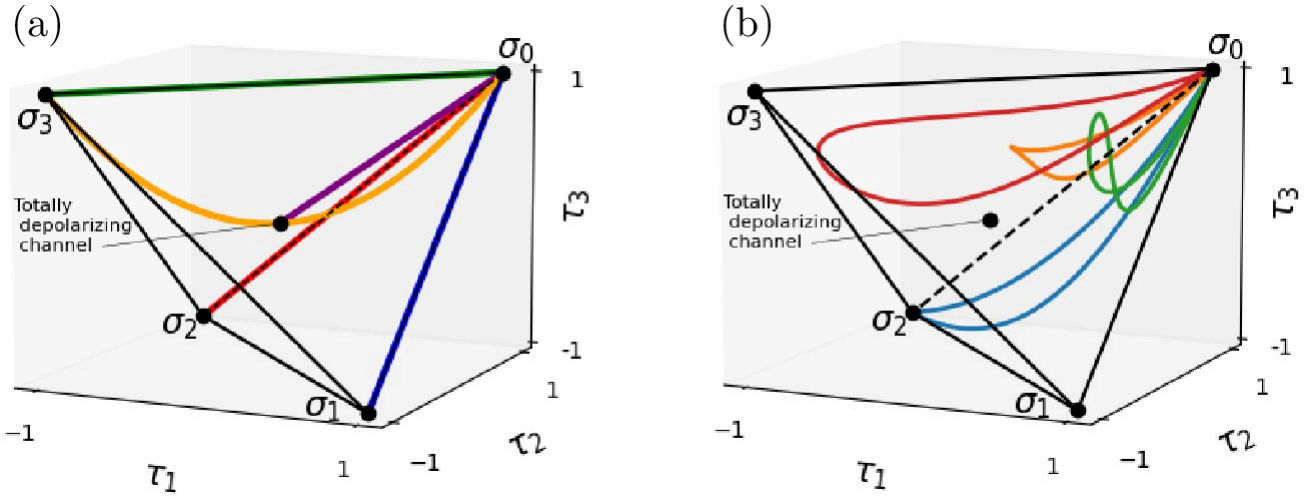
Some Pauli dynamical maps that can be implemented with a 1PR circuit. The curves painted in these tetrahedrons represent Pauli dynamical maps that can be implemented with 1PR circuits. (a) shows the dynamical maps mentioned in the main text, which are: depolarizing (purple), bit flip (blue), phase flip (green), bit-phase flip (red) and parabolic (orange). (b) shows dynamical maps selected at random that can be implemented with 1PR circuits.

For the particular case of one qubit, we can show some examples of Pauli dynamical maps implementable with a 1PR circuit, which are plotted in Fig 6. The examples we show include some of the most common maps: the bit flip, phase flip, bit-phase flip and depolarizing. However, we also include the parabolic dynamical map, defined in Eq (32) and shown in Fig 6. This map traces a parabola inside the tetrahedron connecting two of its vertices and it describes a frontier in the tetrahedron between Pauli channels that are reachable by Lindbladian dynamics and those that are not [19].

**Fig 7:**
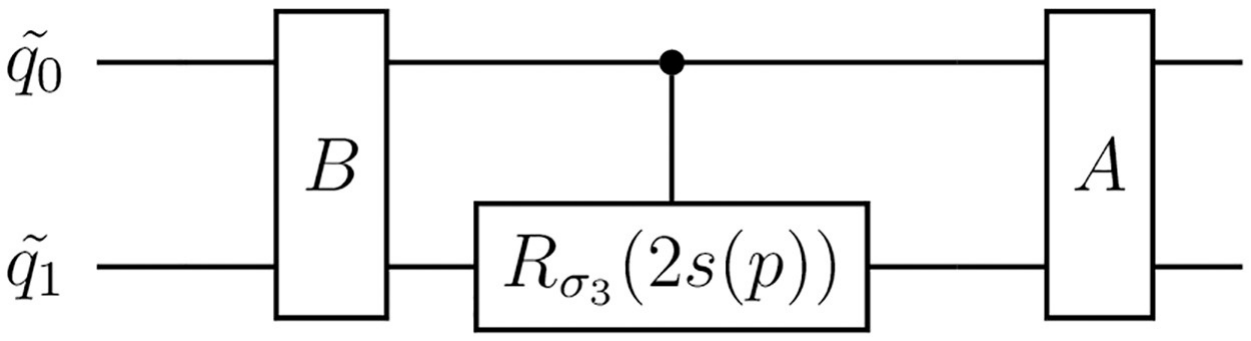
1PR Circuit for one qubit curve of states. This is a 1PR circuit for creating a curve of states that satisfies the conditions of theorem 2. given the curve of states, operations *A* and *B* can be defined as in Eq (28).

**Fig 8:**
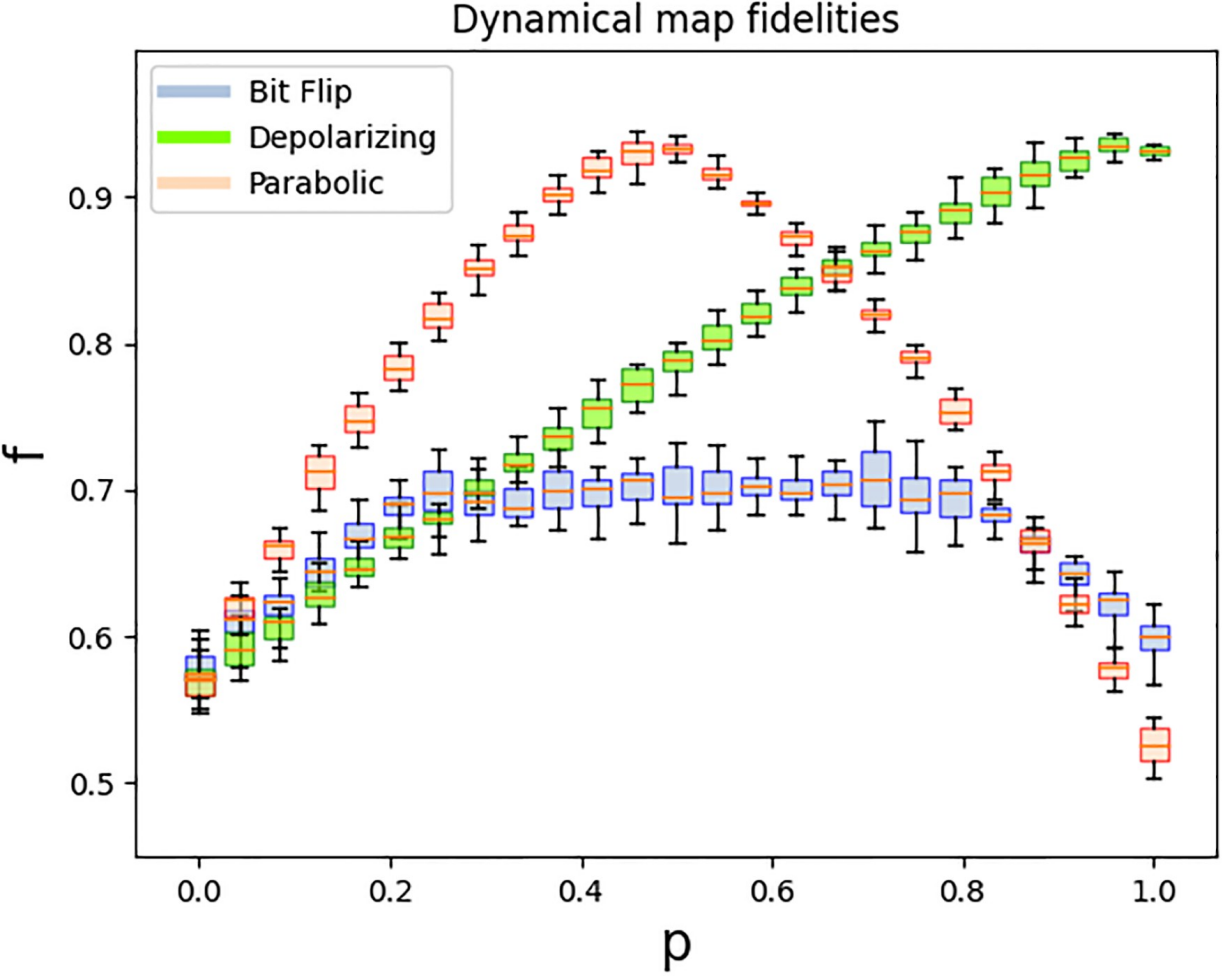
Fidelities for dynamical maps. Results of the fidelities obtained when simulating the three dynamical maps considered (depolarizing, parabolic and bit flip). The implementation was done on IBM’s ibmq-lima quantum computer. Furthermore, we can see that the fidelities are comparable to those of Fig 3, so that using the 1PR circuit doesn’t considerably affect fidelity.

- **Depolarizing:** This dynamical map is given by
  $$\begin{array}{c} \varepsilon_{p}\left(\rho \right)=\left(1-3p/4\right)\rho +\left(p/4\right)\sigma_{1}\rho \sigma_{1}+\left(p/4\right)\sigma_{2}\rho \sigma_{2}+\left(p/4\right)\sigma_{3}\rho \sigma_{3}, \end{array}$$ (31)
  with *p* ∈ [0, 1]. Therefore, the curve of states |*β*(*p*)〉 needed on the ancilla qubits is such that |*β*_0_(*p*)|^2^ = (1 − 3*p*/4), |*β*_1_(*p*)|^2^ = |*β*_2_(*p*)|^2^ = |*β*_3_(*p*)|^2^ = *p*/4. Then, taking the *β*_*j*_ to be real, the curve of states can be
  $$\begin{array}{c} \left|\beta \left(p\right)\right\rangle =\sqrt{1-3p/4}\left|0\right\rangle +\sqrt{p/4}\left|1\right\rangle +\sqrt{p/4}\left|2\right\rangle +\sqrt{p/4}\left|3\right\rangle . \end{array}$$
  This state can be rewritten as:
  $$\begin{array}{cc} |\beta (p)\rangle = & \;e^{is}(\frac{1}{2}\left|0\right\rangle -\frac{i}{2\sqrt{3}}\left|1\right\rangle -\frac{i}{2\sqrt{3}}\left|2\right\rangle -\frac{i}{2\sqrt{3}}\left|3\right\rangle )\\ & +e^{-is}(\frac{1}{2}\left|0\right\rangle +\frac{i}{2\sqrt{3}}\left|1\right\rangle +\frac{i}{2\sqrt{3}}\left|2\right\rangle +\frac{i}{2\sqrt{3}}\left|3\right\rangle )\\ \equiv & \;e^{is}\left|a\right\rangle +e^{-is}\left|b\right\rangle \end{array}$$
  with $\text{sin}\;s=\sqrt{3p/4}$. We can see that this curve satisfies the conditions of Eq (27), meaning that it can be created with a 1PR circuit. Explicitly, following the discussion after theorem 2, we know that this curve of states can be constructed by the circuit of Fig 7.
  The circuit requires defining *A* and *B* as in Eq (28), and for the particular vectors |*a*〉, |*b*〉, |*c*〉 of this dynamical map, we have that
  $$\begin{array}{c} A=(\begin{array}{cccc} 0 & 0 & 1/\sqrt{2} & 1/\sqrt{2}\\ 1/\sqrt{6} & -1/\sqrt{2} & i/\sqrt{6} & -i/\sqrt{6}\\ 1/\sqrt{6} & 1/\sqrt{2} & i/\sqrt{6} & -i/\sqrt{6}\\ -\sqrt{2/3} & 0 & i/\sqrt{6} & -i/\sqrt{6} \end{array}),\;B=(\begin{array}{cccc} 0 & 0 & 0 & 1\\ 0 & 0 & 1 & 0\\ 1/\sqrt{2} & -1/\sqrt{2} & 0 & 0\\ 1/\sqrt{2} & 1/\sqrt{2} & 0 & 0 \end{array}), \end{array}$$
  where, as mentioned before, the first column of *A* and the last three of *B* can be chosen arbitrarily as long as the resultant matrices are unitary. We ran this circuit in IBM’s quantum computer ibmq-lima for 25 different values of *p* between 0 and 1, with 20 repetitions for each value of *p*. The diamond fidelity was calculated in each case and is shown in Fig 8.
- **Parabolic dynamical map:** We define the parabolic dynamical map as:
  $$\begin{array}{c} \epsilon \left(\rho \right)={\left(1-p\right)}^{2}\rho +\left(p-p^{2}\right)\sigma_{1}\rho \sigma_{1}+\left(p-p^{2}\right)\sigma_{2}\rho \sigma_{2}+p^{2}\sigma_{3}\rho \sigma_{3}, \end{array}$$ (32)
  with *p* ∈ [0, 1]. If we take the *β*_*j*_ to be real, the curve of states needed on the ancilla qubits can be:
  $$\begin{array}{c} \left|\beta \left(p\right)\right\rangle =\left(1-p\right)|0\rangle +\sqrt{p-p^{2}}\left|1\right\rangle +\sqrt{p-p^{2}}\left|2\right\rangle +p|3\rangle . \end{array}$$ (33)
  This can be rewritten as
  $$\begin{array}{cc} |\beta (p)\rangle = & \;(\frac{1}{2}\left|0\right\rangle +\frac{1}{2}\left|3\right\rangle )+e^{is}(\frac{i}{4}\left|0\right\rangle +\frac{1}{4}\left|1\right\rangle +\frac{1}{4}\left|2\right\rangle -\frac{i}{4}\left|3\right\rangle )\\ & +e^{-is}(\frac{-i}{4}\left|0\right\rangle +\frac{1}{4}\left|1\right\rangle +\frac{1}{4}\left|2\right\rangle +\frac{i}{4}\left|3\right\rangle )\\ \equiv & \;|c\rangle +e^{is}\left|a\right\rangle +e^{-is}\left|b\right\rangle , \end{array}$$
  with sin *s* = 2*p* − 1, so that this curve fulfills the conditions of Eq (27). This means that it can be created with the 1PR circuit of figure Fig 7. In this case, matrices *A* and *B* can be taken to be:
  $$\begin{array}{c} A=(\begin{array}{cccc} 0 & 1/\sqrt{2} & -i/2 & i/2\\ -1/\sqrt{2} & 0 & 1/2 & 1/2\\ 1/\sqrt{2} & 0 & 1/2 & 1/2\\ 0 & 1/\sqrt{2} & i/2 & -i/2 \end{array}),B=(\begin{array}{cccc} 0 & 1 & 0 & 0\\ 1/\sqrt{2} & 0 & 0 & -1/\sqrt{2}\\ 1/2 & 0 & 1/\sqrt{2} & 1/2\\ 1/2 & 0 & -1/\sqrt{2} & 1/2 \end{array}), \end{array}$$
  where again, the first column of *A* and the last three of *B* can be chosen arbitrarily. As before, we ran this circuit for 25 values of *p* between 0 and 1, with 20 repetitions for each, obtaining as a result the fidelities shown in Fig 8.
- **Bit flip map:** This dynamical map is defined as
  $$\begin{array}{c} \varepsilon_{p}\left(\rho \right)=\left(1-p\right)\rho +p\sigma_{1}\rho \sigma_{1}, \end{array}$$
  for *p* ∈ [0, 1]. In particular, if we take the *β*_*j*_ to be real, we need to create the curve of states:
  $$\begin{array}{c} \left|\beta \left(p\right)\right\rangle =\sqrt{1-p}\left|0\right\rangle +\sqrt{p}\left|1\right\rangle . \end{array}$$
  This can be rewritten as
  $$\begin{array}{c} \left|\beta \left(p\right)\right\rangle =e^{is}(\frac{1}{2}\left|0\right\rangle -\frac{i}{2}\left|1\right\rangle )+e^{-is}(\frac{1}{2}\left|0\right\rangle +\frac{i}{2}\left|1\right\rangle ), \end{array}$$
  with $\text{sin}\;s=\sqrt{p}$. Therefore, we can see that the curve satisfies the conditions of Eq (27) and it can be created with a 1PR circuit. Matrices *A* and *B* can be obtained as for the other dynamical maps and we ran the resulting circuit in ibmq-lima with the same specifications as for the other examples, with the results are shown in Fig 8.
  The exact same thing can be done for the phase flip and bit phase flip dynamical maps by changing *σ*_1_ to *σ*_3_ and *σ*_2_ respectively. For example, the bit phase flip map was implemented in [13, 34] using an optical arrangement, and it was indeed done by varying only one angle that depends on the parameter *p* (the angle of a half waveplate).

From the results of Fig 8, we can see that just as in figure Fig 3, fidelities are high for points close to the depolarizing channel (*p* = 1 for depolarizing map, *p* = 1/2 for parabolic map) and lower for points close to unitary channels (*p* = 0 for all maps and *p* = 1 for parabolic and bit flip). Furthermore, we can see that the actual values of the fidelities are comparable to those of Fig 3, which means that using the 1PR circuit as opposed to implementing each channel with Fig 2 does not considerably affect fidelity.

Furthermore, we can construct other examples of Pauli dynamical maps such that they can be implemented with a 1PR circuit. To do it, we only need to choose the three states |*a*〉, |*b*〉, |*c*〉 that satisfy the conditions of Eq (27). For example, this can be done systematically for the case of curves of states of two qubits (that is, for Pauli dynamical maps of one qubit) with the following procedure:

1. We first choose the norms |*a*|, |*b*|, |*c*| such that 〈*a*|*a*〉 + 〈*b*|*b*〉 + 〈*c*|*c*〉 = 1. This can be done by selecting two angles *μ* ∈ [0, *π*/2], *ν* ∈ [0, *π*/2] and defining:
  $$\begin{array}{c} |a|=\text{sin}\;\nu \text{cos}\;\mu ,\;|b|=\text{sin}\;\nu \text{sin}\;\mu ,\;|c|=\text{cos}\;\nu . \end{array}$$
2. We define |*a*′〉 = |*a*||0〉, |*b*′〉 = |*b*||1〉, |*c*′〉 = |*c*||2〉.
3. Finally, we choose a unitary matrix *V* with the condition that its first row is equal to *e*^*iθ*^(|*a*|, |*b*|, |*c*|, 0) with *θ* a uniform random phase. That way, we can define |*a*〉 = *V*|*a*′〉, |*b*〉 = *V*|*b*′〉, |*c*〉 = *V*|*c*′〉 and since *V* is unitary, these unprimed vectors will fulfill the conditions of Eq (27). Furthermore, the form of the first row ensures that the dynamical map begins at the identity, since it implies that when *s* = 0, the state created in Eq (30) is |*a*〉 + |*b*〉 + |*c*〉 = *e*^*iθ*^|0〉, which corresponds with applying the identity channel.
  Such a matrix *V* can be randomly constructed by first finding three vectors ${\vec{w}}_{1},{\vec{w}}_{2},{\vec{w}}_{3}$ orthogonal to the first row using the Gram-Schmidt process. Then selecting random complex numbers *r*_1_, *r*_2_, *r*_3_ such that |*r*_1_|^2^ + |*r*_2_|^2^ + |*r*_3_|^2^ = 1 and defining the second row of *V* to be $r_{1}{\vec{w}}_{1}+r_{2}{\vec{w}}_{2}+r_{3}{\vec{w}}_{3}$. Once the first two rows are chosen, use Gram-Schmidt to find two vectors ${\vec{v}}_{1},{\vec{v}}_{2}$ orthogonal to them and similarly define the third row as $q_{1}{\vec{v}}_{1}+q_{2}{\vec{v}}_{2}$ with |*q*_1_|^2^+ |*q*_2_|^2^ and *q*_1_, *q*_2_ selected at random. Finally, there is only one choice for the fourth row so that it is orthonormal to the first three and a random phase can be given to it.

Following this procedure for random angles and unitary matrices *V*, we plot four Pauli dynamical maps selected at random that can be implemented with a 1PR circuit in Fig 6.

## 6 Conclusion

In this work, we found a quantum algorithm for simulating Pauli channels in *N*-qubit systems and generalized it to Pauli dynamical maps by using parametrized quantum circuits. Furthermore, we implemented single-qubit Pauli channels on one of IBM’s quantum computers and obtained their fidelities.

Then, when working with Pauli dynamical maps, we searched for a way of minimizing the amount of parametric operations in the circuit by requiring that only one single-qubit rotation depends on the parameter. In theorem 2 we found the general mathematical conditions for this, applicable to any parametrized circuit. Given this condition, we considered the space of Pauli maps that are possible to parametrize with one parameter, and gave some explicit examples for a system of one qubit. As the number of qubits gets bigger, the degrees of freedom for choosing a Pauli dynamical map also gets bigger, so we would expect that the space of maps that can be done with only one parameter becomes a relatively smaller part of the whole space of Pauli dynamical maps. However, some paradigmatic maps as the dephasing one we studied for 2 qubits will still be possible to do with a 1PR circuit (for this case, this can be seen by noticing that said dephasing channel has the same form as the depolarizing channel for one qubit, which we proved to be possible to implement with a 1PR circuit).

In conclusion, this work presents yet another example of the current exploration into simulating open quantum systems in quantum computers, and we observe the effect that the error of a quantum computer have on these simulations, quantified by the fidelity. On the other hand, the result of theorem 2 shows what can be done with the condition of using only one parametrized rotation and can be applied to any quantum algorithm that requires parametrized circuits, such as those used for quantum machine learning [21].
